# Anaesthetica

**Published:** 1863-05

**Authors:** 


### Selections.

---

ANÆSTHETICA.—A Sub-Order of the Order *Æsthetica*, or agents which affect sensation. The Anæsthetics, as their name imparts, *obliterate* pain; but the term has also been applied to agents which only *diminish* common sensibility, or the sensibility to pain. More recently, its use has been confined to those agents which have the power of totally suspending the common sensibility of the body.

The humane surgeon has always been desiring something to lessen or extinguish pain; and many things have been resorted to to fulfil that desire. About the year 1830, and even earlier, it was demonstrated that patients could, sometimes, be thrown into what was called the *biological* condition, and while in that condition, surgical and other operations could be performed without the patient's being sensible of pain. About that time, also, Dr. James Arnott attempted to bleed patients to syncope, and to perform surgical operations while the patient remained unconscious from fainting. Several minor operations were performed, and a breast was excised, while the patient remained unconscious from venesection.

In regard to the introduction of the use of the etherial vapors, the profession seems to be still somewhat divided, but the following statement is thought to be substantially correct. Early in the present century, Sir Humphrey Davy published the following: "As *nitrous oxide*, in its extensive operations, seems capable of destroying physical pain, it may probably be used with advantage during surgical operations in which no great effusion of blood takes place." We have no account of this idea having been acted on, until, in 1840, Dr. Horace Wells, a dentist, at Hartford, Connecticut, after witnessing the intoxicating influence of nitrous oxide gas, said, "that he believed that a man might be made so drunk by this gas, or some similar agent, that dental or other operations might be performed on him without any sensation of pain on the part of the patient."

In the winter of 1841–1842, as stated by Prof. Charles T. Jackson, he mentioned to his friends that he had discovered and tried upon his own person, the anæsthetic power of sulphuric ether, and during that year he mentioned the fact to five or six of his friends. In 1844 Dr. Horace Wells again remarked, that he believed a man, by taking nitrous oxide gas, could have a limb amputated and not feel the pain. On September the 11th, 1844, at the request of Horace Wells, Dr. J. M. Riggs was asked to extract a tooth for a gentleman who had inhaled nitrous oxide gas, and who said, "It did not hurt me more than the prick of a pin." This is the earliest account we have of any surgical operation having been performed upon a patient while under the influence of an anæsthetic etherial vapor. In 1846, Dr. William F. Channing, an assistant to Prof. Charles T. Jackson, came near losing his life by the accidental inhalation of chlorine gas. Prof. Jackson at once had him inhale the vapor of sulphuric ether, which gave immediate relief to the excruciating pain in his lungs and throat.

But, at least two years previously, in the fall of 1844, Dr. Horace Wells had delivered a lecture to the medical class of Dr. Warren in the Massachusetts General Hospital, on "Rendering the system insensible to pain during the inhalation of exhilarating gas." For some reason, no attempt was made at the time to perform an operation upon a patient who had inhaled an anæsthetic.

*Sulphuric Ether* had been used by inhalation, (see *Sulphuric Ether.*) almost from its discovery. In 1795, Dr. Thornton spoke of the relief from pain which followed its inhalation. But it was not until the fall of 1846, (October 16 and 17,) that a full and public series of experiments were made, demonstrating the anæsthetic power of Sulphuric Ether, under the management of a Mr. Morton, dentist, with the advice of Dr. Jackson, at the Massachusetts General Hospital, when surgical operations were performed on insensible patients by Drs. Warren and Hayward. In two or three weeks other operations were performed on patients rendered insensible by inhaling the vapor of Sulphuric Ether; and from that date *Anæsthesia* preparatory to important surgical operations, may be considered as fully established. Before the close of the year, anæsthesia had been tested in London, (Dec. 19.) On the 12th of January, 1847, Malgaigne reported the results of several operations under its use; and in a remarkably short time it was tested in all the principal cities in Europe.

Since the introduction of Anæsthesia, a very large number of agents have been found to be capable of destroying sensibility to pain, to be true Anæsthetics; and the profession have endeavored to decide upon those which are most desirable for use under special circumstances. The most important of these are carbonic acid, (see *Acid Carbonic*,) Chloroform, (which see,) Cold, (see *Cold*,) and the Ethers, (see *Ether* and *Sulphuric Ether*.) The most common and the most important of the Anæsthetics in use, at the present time, are Chloroform and Sulphuric Ether; and as the Anæsthetic vapors all have a similarity of action, when inhaled, such general remarks as are applicable to them all are properly inserted in this place.

Anæsthetic vapors, when inhaled, are absorbed into the blood, and from the blood they pass to the nerves and nerve centers, and other organs, which they thus influence by direct contact. Coming in contact with the nerves, these vapors disturb, suspend, and, when long applied, may even destroy the functions of the organs with which they were brought in contact.

All the nerves do not have their functions simultaneously disturbed, suspended, or destroyed by an Anæsthetic; but the nerves become successively affected, as the vapor is carried to them, and thus the functions of the nerves are subdued. A very large amount of blood is carried to the brain, and the power of regulating muscular action and motion, and the power of general sensibility, or of feeling pain, is often lost, while the intellect remains almost unaffected. The ganglionic system of nerves, which control the action of the heart, lungs, and the digestive organs, continues comparatively unaffected, even after a fatal amount of Anæsthetic has been inhaled; and it is only by the direct action of the vapor upon the heart or the lungs that those organs are paralyzed by its action.

The analogy between the action of the Ethers, when inhaled, and the *Inebriants*, including the Ethers, when drank into the stomach, is very close. In fact, the only difference between the *Anœsthetics* and *Inebriants*, of which the Anæsthetics may properly be considered a Sub-Order, consists in the comparative rapidity of the action of the Anæsthetics. These vapors first stimulate, then derange the intellect, then depress all the functions of the brain. The brain, after the momentary exhaltation, first loses its power of governing the organs and function of locomotion, and yet the mind remains unclouded, but sensation is somewhat deranged. Soon, the mind becomes affected, while the external organs of sense are obtunded, and the patient dreams and has visions. When the third stage is reached, respiration continues, there may be motion, (involuntary,) of the muscles, the patient may groan, or give utterance to violent out-cries of pain, but does not articulate words, or give other evidence of mental activity. In this condition most of the slighter surgical operations can be performed without pain and with safety to the patient. When the patient is placed still more under the influence of an Anæsthetic, he becomes entirely insensible, motionless, (except of the lungs and the heart,) the breathing becomes slightly stertorous, the eyeballs and irides fixed. This is the stage of Anæsthesia into which most patients are thrown for grave operations, and for operations about the eye. If more of the Anæsthetic vapor is allowed to be inhaled, after the patient is in the condition last described, than escapes through the breath and through the skin, so that still more of the vapor accumulates in the system, the heart and the lungs may become paralyzed and the patient die. Similar results may also follow, so far as the heart and the lungs, and a fatal termination are concerned, if the vapor is not sufficiently diluted with atmospheric air.

During the first brief stage, that of exhaltation, the patient will frequently exclaim that *he* cannot be made insensible, and that the attempt is useless. During the second stage, he may say that he is not insensible to pain, when in reality he has lost all feeling. The third stage need not be reached in all cases.

When the patients are recovering from the influence of an Anæsthetic, they often acquire the use of all their special senses while their nerves of general sensibility still remain benumbed; and they are hence insensible to pain, even while the intellect remains unaffected sufficiently to allow the patient to fully understand the various slips of an operation.

The propriety of using any form of Anæsthetics in certain forms of disease has been thoroughly discussed, until the profession have settled the matter, for the present. But of the superior value of one of the Ethers over others, there still remains some agitation and doubt. Under the present head, it is not thought best to enter upon a discussion of the merits of the various Anæsthetics, but to leave that to be considered when the value and use of the separate ones are under examination.

Anæsthetics may be used, *in surgical operations*, whenever the pain of the operation would cause very great suffering to the patient, or would cause a severe shock to the nervous system, or lead to such motions or muscular action on the part of the patient, as would embarrass the operator. Anæsthetics should be administered whenever an operation is to be performed, which, without them, would be inexpedient or impracticable:—Whenever their use will afford relief to both patient and surgeon:—Whenever their use, by keeping the patient quiet, and thus allowing the quick performance of a grave operation, will prevent dangerous hemorrhage:—Whenever by relaxation of muscles, they favor reduction of dislocation of joints, or of the tumor of a hernia:—Whenever their use is demanded to save the feelings of modesty in women.

As a general rule, Anæsthesia should *not* be resorted to:—When the patient is affected with a grave disease of the lungs or heart:—When there is disease of the substance of the brain:—When there is special tendency to hemorrhage:—When the sensations of the patient are needed to guide the surgeon in his operations, as in those about the bladder and the uterus:—When the blood from the parts operated upon would pass into the lungs, over the paralyzed trachea or bronchiæ, as in the operations upon the palate, the uvula, the tonsils, or the wind-pipe.

Anæstheia may be resorted to in *midwifery*:—In all ordinary cases, where not contra-indicated by any of the conditions enumerated, whenever the patient greatly desires it:—In all unusually painful, laborious, or complicated labors:—When there is great tenderness of the parts concerned in labor:—When there is great rigidity of the os uteri, the vagina, or the vulva:—When there is great nervous irritability, with anxiety or apprehension:—Whenever any considerable manual or instrumental interference is required:—Whenever there are puerperal convulsions, uræmic or others:—In those cases of inefficient uterine action, where prolonged labor, loss of sleep, or great mental anxiety, have greatly interfered with the progress of labor:—In cases of adherent placenta, where it is necessary to introduce the hand and separate the adhesions:—In cases of extensive laceration of the perineum, especially if sutures must be inserted to keep the torn parts in coäptation.

Anæsthetics are of incalculable value in the practice of obstetrics.

When properly administered, they exert no injurious influence upon either mother or offspring.

It is entirely proper to use Anæsthetics in natural labor, simply for the purpose of lessening the pain of the mother.

Anæsthetics are of very great value in calming the nervous agitation and mental anxiety of the parturient female.

In cases where the pains are irregular, uncertain, unsatisfactory and inefficient, or intense and constant, the Anæsthetics are exceedingly useful.

In various forms of *Neuralgia*, and in *Tetanus*, the anæsthetic vapors, inhaled, will afford prompt and sometimes permanent relief. *Hysteria*, in all its myriad forms, yields more readily to Anæsthetics than to any other class of agents. *Puerperal Convulsions*, and *Infantile Convulsions*, concomitant with teething, almost immediately yield to the administration of the Anæsthetic vapors. *Whooping Cough*, and *Spasmodic Asthma*, as well as *Mania* and *Delirium Tremens*, are often relieved almost immediately by the inhalation of an Anæsthetic. *Dysmenorrhœa*, the passage of biliary and urinary *calculi*, and all forms of sudden *cramp* or spasmodic contractions of muscles, are relieved by Anæsthetics. All forms of feigned diseases, phantom tumors, suspected or pretended pregnancies, lameness or immovable joints, soon reveal what they actually are, when the subject is placed under the full influence of an Anæsthetic.

Anæsthesia has been resorted to to allay the vomiting of pregnant women, or the allied vomiting caused by dislocation and inflammation of the uterus—to lessen aggravated after pains—to cause sleep in delirium tremens, and the exhausting delirium, and loss of sleep in continued fever—to prevent the ague fit in fever and ague—to break up the paroxysms in whooping cough—to relieve spasmodic asthma—to afford relief to that form of insanity of the muscles called chorea—to relieve puerperal mania—to control the agony of angina pectoris, or neuralgia of the heart—in various forms of diseases of the eye—in infantile convulsions, hysterical convulsions, and other convulsive diseases—and, in short, whenever, in the entire range of diseases, their use appeared to be indicated. And, while they have afforded relief in millions of cases, their use seems to have been most wonderfully exempt from all danger except that which has been caused by carelessness and recklessness of administration.

For the special uses of the various agents belonging to this Sub-Order, and for directions in regard to their administration and use, see *Chloroform*, *Sulphuric Ether*, and *Cold*.

*Journal of Rational Medicine.*

